# Supplementary material for: Chromothripsis during telomere crisis is independent of NHEJ, and consistent with a replicative origin
Source: Genome Res. 2019 May;29(5):737–49. doi: 10.1101/gr.240705.118 (PMC6499312; doi:10.1101/gr.240705.118)
Supplement: Supplemental Material [file supp_gr.240705.118_Supplemental_file_1.zip › contigs/annotated_contigs/DB96/contig.2.DB96_length_1032_mean_cov_36.3837209302.docx]

**DB96_length_1032_mean_cov_36.3837209302**

```
AAAAGGAGG|AAACAAAAAGAAAGAAAGAGAAA|AAAAGGGA|GAAGGAAAAAAAGACAGAAAGAGAGAAAGAAGGAAAGGAAGAGAGG
          >chr10:21644451-21644474 + E=7e-02 p=0e+00  >chrUn_gl000220:102865-103869 -
AAGAAAAACCTAAAGAAGGAGAGAAAGGAAGAAAGGAAGGAAGAAAAACACGAAGGAGAGGAAGAAAGAAAACAGAGATAACTACGTAC
E=0e+00
GCTCGTTCATTTACACACATAAATACGACGCTTTTCATACGTAAAATAAACGTCTTTATCGACGATCCCTTCTTTATAGAGCGATGTGT

ATTTATTTGTATAACACAGACACCTACATCTATCATACAGAAGTCTATTTCCATACAACCGATACGTATTTACCATACGCAAGAGTATT

CAATGCAGAGATACACGTTGTCGTTGTTTGCATATAAGCGTACAGAAACGTTTACATTAATACATATAAGTAAACGCGTGGAAACGAAA

GAAATAAAAAAGCGAAATGAGTCAACAGGCCGGGCACGGTGGCTCACGCCCGTCATCCCAGCACTTCGAGAGGCCGAGGTGGGCGCATC

ACAGGAGGTCGGGAGTTGGAGACCAGCCTGAGCAACATGGAGAGACACGGCGTGCCTACTAAAAACACAAACATCAGCCAAGCCAGGCG

TGGGGGTGCCTCCCTGTCATCCCCGCTAATCGGGAGGCTGAGGCAGGAGAAGCGCTCGAACCCGGGAGGCGGAAGGTGCGGTGAGCCAA

GACCGCGCCATTGCACTCTAGCCGTGGAAACAAGAGTGAAACTCTGTCTCAAAAGGACGAAACAGAAAGAAAGAAAGAAAGGAAGGAAG

GAAGGAAGGAAGGAAGGAAGAAAGAAAGAAAAGAAAGAAGGAATGAATGAAAGAAAAGAAAGCAAGAAAGAAAGAAAGAAAGAAAAAGA

AAAGAAAGAAAGAAAAGAAAGCAAGAAAGCACGAAAGCAAGCAAGCAAGAAAGCAAGAAAACAAGGAAGCAAGAAAGCAAGCAAGAAAG

AAACAAAAGAAAGAAAGCAAGAAAACAAGAAAGCACGAAAGCAAGCAAGCAAGAAA
```
